# Circular RNA drives resistance to anti-PD-1 immunotherapy by regulating the miR-30a-5p/SOX4 axis in non-small cell lung cancer

**DOI:** 10.20517/cdr.2021.100

**Published:** 2022-03-25

**Authors:** Jing Wu, Meng-Xuan Zhu, Ke-Sang Li, Ling Peng, Peng-Fei Zhang

**Affiliations:** ^1^Department of Medical Oncology, Zhongshan Hospital, Fudan University, Shanghai 200032, China.; ^2^Cancer Center, Zhongshan Hospital, Fudan University, Shanghai 200032, China.; ^3^Department of Hematology and Oncology, Hwa Mei Hospital, University of Chinese Academy of Sciences, Ningbo 315012, Zhejiang, China.; ^4^Department of Pulmonary and Critical Care Medicine, Zhejiang Provincial People’s Hospital, Hangzhou 310014, Zhejiang, China.; ^#^Authors contributed equally.

**Keywords:** NSCLC, immune evasion, circRNAs, PD-1, immunotherapy resistance

## Abstract

**Aim: **Circular RNAs are widely and abnormally expressed in human cancer cells, and they participate in cancer progression. However, they have rarely been investigated in the immune evasion of non-small cell lung cancer (NSCLC). Here, we elucidated the function and molecular mechanism of hsa_circ_0020714 in promoting the resistance to anti-PD-1 immunotherapy of NSCLC.

**Methods: **The expression of hsa_circ_0020714 were examined by qRT-PCR. *In vivo *experiments were executed to investigate the biological function of hsa_circ_0020714 in the sensitivity of NSCLC to anti-PD-1 immunotherapy. The qRT-PCR, fluorescence *in situ *hybridization, RNA pulldown, RNA immunoprecipitation, and western blot were carried out to investigate the potential regulatory mechanisms of hsa_circ_0020714 in NSCLC immune evasion.

**Results: **The expression of hsa_circ_0020714 was upregulated in NSCLC tissues compared to the paired adjacent non-tumor tissues, and an increased expression of hsa_circ_0020714 was significantly associated with a bad prognosis and resistance to anti-PD-1 immunotherapy in patients with NSCLC. Mechanistically, hsa_circ_0020714 functions as an endogenous miR-30a-5p sponge to enhance SOX4 expression, thereby promoting immune evasion and anti*-*PD*-*1 resistance in NSCLC patients.

**Conclusion: **Hsa_circ_0020714 induces the immune evasion and resistance to anti-PD-1 immunotherapy of NSCLC via the miR-30a-5p/SOX4 axis, and may be an promising immunotherapeutic target in NSCLC.

## INTRODUCTION

Advanced non-small cell lung cancer (NSCLC) with a negative driver gene has a high tendency for distance metastatic and resistance to chemotherapy^[1,2]^. Recent evidence suggests a critical role for anti-programmed death-1 (anti-PD-1) immunotherapy in driver gene negative NSCLC. Although anti-PD-1 immunotherapy has improved the prognosis of patients with advanced NSCLC, a larger proportion of patients still did not respond to anti-PD-1 therapy due to primary or secondary resistance and failed to demonstrate clinically effective responses^[3,4]^. Therefore, further investigation is necessary to clarify the molecular mechanisms involved in NSCLC immune evasion and develop new immunotherapeutic approaches for patients with NSCLC.

Circular RNAs (circRNAs) are recently a class of novel non-coding RNAs (ncRNAs) with circular configurations and have attracted much attention^[5,6]^. With the rapid development of RNA sequencing (RNA-Seq) technology, circRNAs have been confirmed to be dysregulated frequently in most cancers, and to play critical regulatory roles in the process of tumorigenesis and progression^[7]^. Several dysregulated circRNAs have been reported to be involved in NSCLC cell proliferation, invasion, migration, immune evasion, and chemotherapy resistance, for example circFGFR1^[8]^, circNDUFB2^[9]^, and circMET^[10]^. SOX4 is a member of the SOX (Sry-related high-mobility group box) transcription factor family^[11]^. The *SOX4 *gene is frequently amplified and overexpressed in most malignancy tumors. Evidence increasingly demonstrates that SOX4 act as an oncogene in several cancer progression^[12]^. Bagati *et al.*^[13] ^reported that increased expression of SOX4 induced immune evasion and promoted resistance to anti-PD-1 immunotherapy in triple-negative breast cancer.

Here, we reported a novel cancer immune evasion-related circRNA hsa_circ_0020714, which is significantly upregulated in NSCLC tissues compared with nontumor adjacent tissues. And forced expression of hsa_circ_0020714 is associated with immune evasion of NSCLC and predicts a poor prognosis. Functionally, knockdown and overexpression experiments indicated that hsa_circ_0020714 regulates the sensitivity of NSCLC to anti-PD-1 immunotherapy. Moreover, hsa_circ_0020714 acts as a sponge for miR-30a-5p to upregulate SOX4 expression and consequently induces NSCLC immune evasion. This study reveals an innovative new insight into the underlying molecular mechanism of NSCLC immune evasion and sheds light on hsa_circ_0020714 as a promising sensitivity prediction biomarker and a potential therapeutic target for NSCLC immunotherapy.

## METHODS

### Cell culture

Human NSCLC cell lines (A549, HCC827, NCI-H1299, NCI-H1650, NCI-H226, and NCI-H460) and HEK-293T cells were purchased from Cell Bank of the Chinese Academy of Sciences (Shanghai, China). These cells were routinely cultured in DMEM medium (HyClone, Logan City, USA) supplemented with 10% fetal bovine serum (Gibco, Carlsbad, USA) and 1% penicillin/streptomycin (100 IU/mL) at 37 °C in a humidified incubator with 5% CO_2_.

### Patients and tissues

We obtained NSCLC tissues and matched adjacent non-tumoral tissues from patients who underwent surgery at the Zhongshan Hospital of Fudan University and Second Affiliated Hospital of Nanchang University. The tissues from NSCLC patients were counterstained with hematoxylin and eosin and were confirmed independently by two independent pathologists. All patients or their guardians gave written informed consent for the use of their samples before collection. This study received approval from the Ethical Review Committee of the Zhongshan Hospital of Fudan University.

### Total RNA extraction and quantitative real-time polymerase chain reaction detection

Total RNA extraction and quantitative real-time polymerase chain reaction (qRT-PCR) analysis were performed according to our previous studies^[8,14,15]^. In brief, total RNA from NSCLC tissues, matched adjacent non-tumor tissues, and NSCLC cell lines were extracted using TRIzol (Invitrogen, USA) reagent and then reverse-transcribed into cDNA using a PrimeScript RT Reagent Kit (TaKaRa, Japan). Finally, qRT-PCR was carried out with SYBR Green Real-time PCR Master Mix (Yeasen, Shanghai, China) following the manufacturer’s instructions.

### Mice xenograft anti-PD-1 therapy study, western blot, circRNA immunoprecipitation, RNA pull-down, and dual luciferase reporter assays

Mice xenograft anti-PD-1 therapy study, western blot, circRNA immunoprecipitation (circRIP), RNA pull-down, and dual luciferase reporter assays were evaluated with SPSS software (19.0, SPSS, Inc., Chicago, IL) as described in our previously studies^[8,10,14]^. Experimentation on *C57BL/6 *mice were approved by the Animal Experimentation Ethics Committee of Zhongshan Hospital, Fudan University. And the antibody used in western blot and circRIP assays were listed as follows: Tubulin (Abcam, ab52623), AGO2 (Abcam, ab186733), and SOX4 (Abcam, ab70598).

### Transfection of lentiviral vectors, plasmids, and miRNA mimics

Transfection of lentiviral vectors, plasmids, and miRNA mimics were evaluated with SPSS software (19.0, SPSS, Inc., Chicago, IL) as described in our previously studies^[10,14]^. In brief, Hsa_circ_0020714 overexpression lentiviral vectors, miR-30a-5p mimics, or pLG3 plasmid containing the sequence of wild type/mutant hsa_circ_0020714/ SOX4 mRNA 3′-UTR were constructed (Shanghai Genomeditech Co. Ltd., Shanghai, China). Transfectant cells were characterized by qRT-PCR or western blotting.

### Statistical analysis

Statistical analysis was conducted using the SPSS software (19.0, SPSS, Inc., Chicago, IL) as described in our previously studies^[10,14]^. *P *< 0.05 was considered statistically significant.

## RESULTS

### Hsa_circ_0020714 is highly expressed in NSCLC tissues and is correlated with poor prognosis

**Figure 1:**
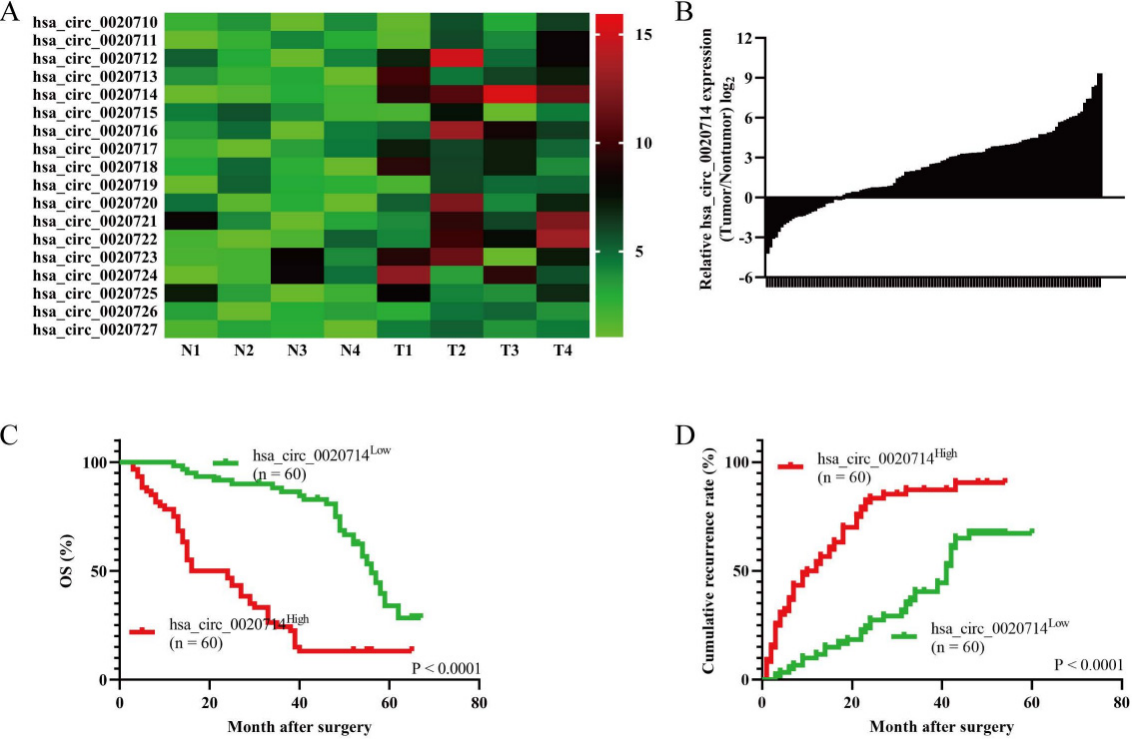
Forced hsa_circ_0020714 expression in the non-small cell lung cancer (NSCLC) tissues correlated with poor prognosis. (A) The heatmap shows *CD151* gene-derived circRNAs in pairs of NSCLC tissues and adjacent nontumor tissues using qRT-PCR. (B) The differential expression of hsa_circ_0020714 in the NSCLC tissues and matched adjacent nontumor tissues of 120 patients, as indicated. (C, D) Prognostic analysis of hsa_circ_0020714 expression in 120 NSCLC patients.

Previous studies have indicated that forced CD151 expression in NSCLC was correlated to poor prognosis^[16]^. Importantly, CD151-derived circRNA circ_0020710 was significantly overexpressed in melanoma tissues and its upregulated expression induced melanoma immune evasion^[17]^. Therefore, we speculated that the dysregulation of CD151-derevied circRNAs may act as oncogenes in NSCLC cells. To explore CD151-derived circRNA expression in NSCLC tissues and matched adjacent nontumor tissues, we examined the CD151-derived circRNAs expression in four pairs of NSCLC tissues and matched adjacent nontumor lung tissues by using qRT-PCR. The results indicated that hsa_circ_0020714 was upregulated in the NSCLC tissues [Figure 1A]. Next, we examined hsa_circ_0020714 expression in the NSCLC tissues and matched adjacent nontumor tissues and found that hsa_circ_0020714 was significantly increased in the NSCLC tissues (tumor/non-tumor ≥ 2) (74/120) [Figure 1B]. Furthermore, our results demonstrated that patients with upregulated hsa_circ_0020714 expression had a worse prognosis than those with lower levels of hsa_circ_0020714 [Figure 1C and D].

### Higher levels of hsa_circ_0020714 expression is correlated with resistance to anti-PD-1 therapy in NSCLC patients

**Figure 2:**
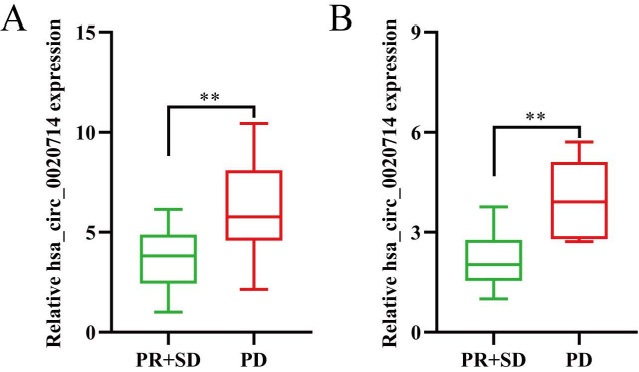
Hsa_circ_0020714 may act as a biomarker for resistance to anti-PD-1 immunotherapy in non-small cell lung cancer (NSCLC) patients. (A) Lung adenocarcinoma. (B) Lung squamous cell carcinoma. **P < 0.01.

Increasingly, studies have reported the dysregulation of circRNAs execute a critical role in cancer immune evasion^[8,18]^. Therefore, we explored whether forced hsa_circ_0020714 expression can decrease the curative effect of anti-PD-1 therapy (Keytruda). Then, we analyzed retrospective data from 42 NSCLC patients (29 cases of lung adenocarcinoma and 13 cases of lung squamous cell carcinoma) with relapse or distant metastasis receiving anti-PD-1 immunotherapy. After six therapy cycles, the efficacy was examined using enhanced computerized tomography and assessed based on iRECIST criterion. For 29 cases of lung adenocarcinoma, the results demonstrated that there were 4 patients with partial response, 14 patients with stable disease, and 11 patients with progressive disease. For 13 cases of lung squamous cell carcinoma, there were 1 patient with partial response, 5 patients with stable disease, and 7 patients with progressive disease. Next, hsa_circ_0020714 expression levels were evaluated via qRT-PCR, and the results demonstrated that the expression levels of hsa_circ_0020714 were higher in the progressive disease group compared with stable disease and partial response groups in both adenocarcinoma and squamous cell carcinoma panels [Figure 2A and B]. These results showed that hsa_circ_0020714 likely participates in the immune evasion and resistance to anti-PD-1 immunotherapy of NSCLC.

### Hsa_circ_0020714 upregulates SOX4 expression sponging miR-30a-5p

**Figure 3:**
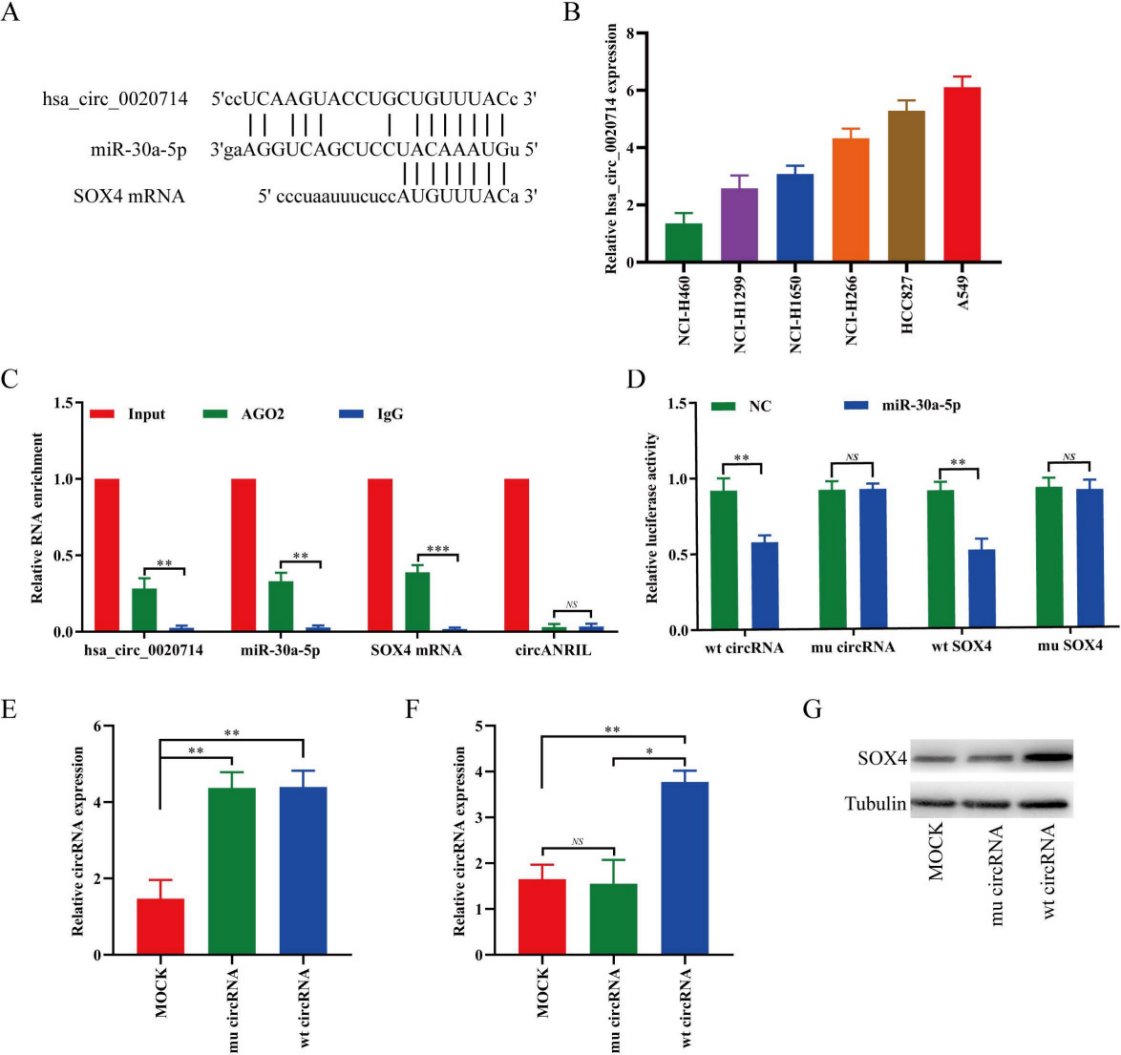
Hsa_circ_0020714 upregulates SOX4 expression via sponging miR-30a-5p in NSCLC cells. (A) Putative miR-30a-5p binding sites to hsa_circ_0020714 and SOX4 were predicated via StarBase v3.0. (B) Hsa_circ_0020714 expression was detected in six NSCLC cell lines using qRT-PCR. (C) CircRIP analysis were carried out using an AGO2 antibody in A549 cells extract. (D) The luciferase activity of wild type (wt) or mutant (mu) pLG3-hsa_circ_0020714/SOX4 mRNA 3′-UTR in the HEK-293T cells after cotransfected with miR-30a-5p. (E) Hsa_circ_0020714 expression was modified in the NCI-H460 cells by lentivirus-mediated cDNA transfection. (F, G) SOX4 mRNA and protein expression levels in wild type (wt) or mutant (mu) hsa_circ_0020714-overexpressing NSCLC cells. ***P < 0.001, **P < 0.01, *P < 0.05. NS: Not significant.

Increasingly, studies have verified that circRNAs act as miRNA sponges. We therefore analyzed whether hsa_circ_0020714 has the ability to induce immune evasion by sponging certain miRNAs. Through StarBase v3.0 analysis, we found that hsa_circ_0020714 and SOX4 were predicated to be possible targets of miR-30a-5p [Figure 3A]. Importantly, it has been reported that forced SOX4 expression promoted immune evasion and resistance to anti-PD-1 immunotherapy in triple-negative breast cancer^[13]^. Based on these results, we speculated that hsa_circ_0020714 promoted NSCLC immune evasion possibly via sponging miR-30a-5p to upregulate SOX4. To further determine the function of hsa_circ_0020714 in immune evasion , we detected hsa_circ_0020714 expression in seven human NSCLC cell lines [Figure 3B]. Next, RIP experiment with argonaute 2 (AGO2) antibody in A549 cells was performed. Our results indicated that hsa_circ_0020714, SOX4 mRNA, and miR-30a-5p, but not circANRIL (a circular RNA that confirmed does not bind to AGO2)^[19]^, were significantly enriched [Figure 3C]. Furthermore, we performed luciferase assays using miR-30a-5p mimics cotransfected with luciferase reporters (which contained a wild-type or miR-30a-5p-target mutant hsa_circ_0020714 sequence/SOX4 mRNA 3′-UTR) into HEK-293 T cells. Compared with the negative control mimics, miR-30a-5p significantly inhibited the luciferase reporter activity in the cells with the wild-type hsa_circ_0020714/SOX4 mRNA 3′-UTR sequence, but not affect the activity in the cells with the miR-30a-5p-target mutant circFGFR1/SOX4 mRNA 3′-UTR sequence [Figure 3D]. To determine whether hsa_circ_0020714 upregulates SOX4 expression via the miR-30a-5p, we established hsa_circ_0020714 or miR-30a-5p target sequence of mutant hsa_circ_0020714 NCI-H460 overexpression cell lines through lentiviral vectors [Figure 3E]. The mRNA and protein levels of SOX4 were significantly increased in NCI-H460 cells following transfection with hsa_circ_0020714, compared with the mock group and mutant hsa_circ_0020714 group [Figure 3F and G].

**Figure 4:**
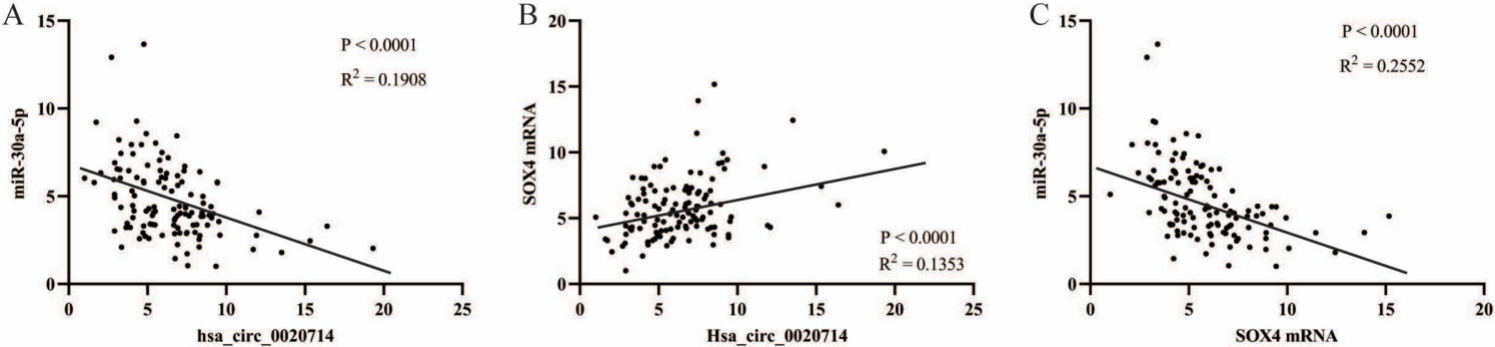
The correlation between hsa_circ_0020714, miR-30-5p, and SOX4 mRNA was analyzed in NSCLC tissues. (A) The correlation between hsa_circ_0020714 and miR-30-5p was analyzed in NSCLC tissues; (B) The correlation between hsa_circ_0020714 and SOX4 mRNA was analyzed in NSCLC tissues; (C) The correlation between miR-30-5p and SOX4 mRNA was analyzed in NSCLC tissues.

**Figure 5:**
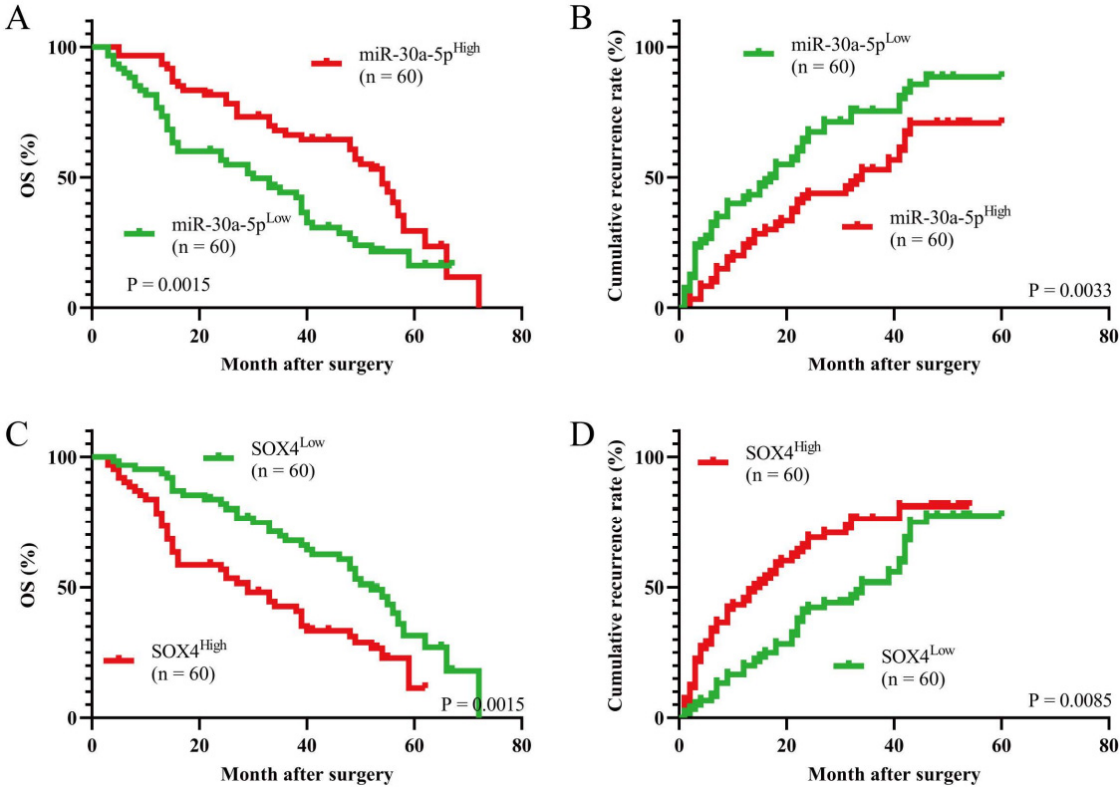
Prognostic analysis of miR-30-5p, and SOX4 mRNA expression in 120 non-small cell lung cancer (NSCLC) patients. (A and B) Prognostic analysis of miR-30-5p expression in 120 NSCLC patients; (C and D) Prognostic analysis of SOX4 mRNA expression in 120 NSCLC patients.

To further verify whether hsa_circ_0020714 upregulates the SOX4 expression by sponging miR-30a-5p, we detected the expression of miR-30a-5p and SOX4 mRNA in above 120 NSCLC patient tumor tissues. The results demonstrated that there was a negative correlation between miR-30a-5p and hsa_circ_0020714/SOX4 mRNA in the NSCLC tissues. Inversely, positive correlations between hsa_circ_0020714 and SOX4 mRNA was observed in the NSCLC tissues [Figure 4A-C]. Moreover, our results demonstrated that patients with upregulated SOX4 mRNA expression had a worse prognosis than those with lower levels of SOX4 mRNA, and patients with increased miR-30a-5p expression had a better prognosis than those with lower levels of miR-30a-5p [Figure 5A-D].

### Hsa_circ_0020714-induced resistance to anti-PD-1 therapy in a SOX4-depedent manner

**Figure 6:**
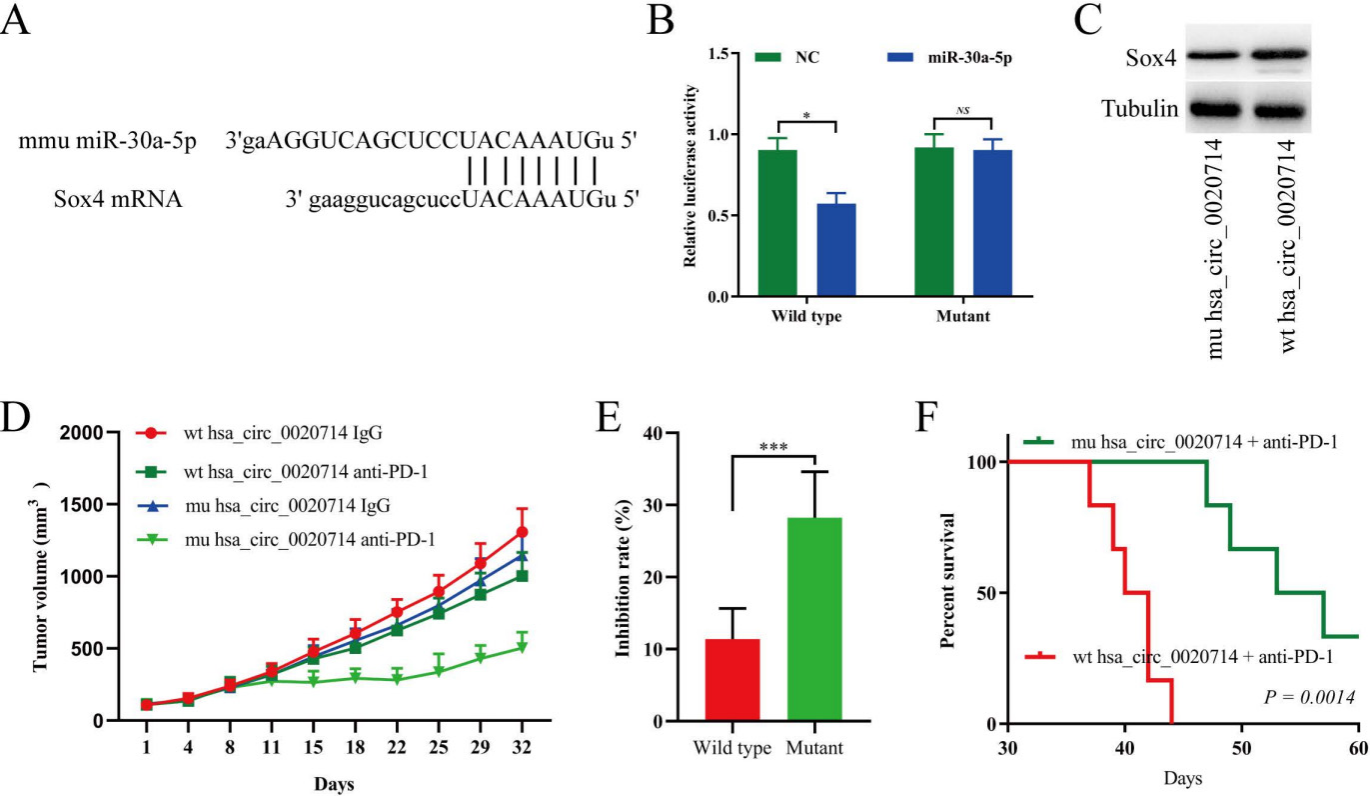
Hsa_circ_0020714 promotes non-small cell lung cancer (NSCLC) resistance to resistance to anti-PD-1 immunotherapy in NSCLC patients. (A) Putative miR-30a-5p binding sites to mouse Sox4 were predicated via StarBase v3.0. (B) The luciferase activity of wild type (wt) or mutant (mu) pLG3-Sox4 mRNA 3′-UTR in the HEK-293T cells after cotransfected with miR-30a-5p. (C) Sox4 expression in the wild type or mutant hsa_circ_0020714-overexpressing cells was detected by western blot. (D) LLC cells with mutant or wild type hsa_circ_0020714-overexpressing were subcutaneously injected into 4-week-old *C57BL/6 *mice. When tumors had reached a mean volume of 100 mm^3^, the mice were treated with an IgG or PD-1 antibody. The data are presented as the mean tumor volume (*n *= 6). (E) The data are expressed as the percentage of tumor growth inhibition (the data are presented as the mean ± SD). (F) The survival curves of the mouse lung xenograft tumors formed by mutant or wild type hsa_circ_0020714-overexpressing cells and treated with a mouse antibody against mouse PD-1. ***P < 0.001, *P < 0.05. NS: Not significant.

Interestingly, humans and mice have the same miR-30a-5p sequence, and mouse SOX4 mRNA 3′-UTR also has a predicted target sequence of miR-30a-5p [Figure 6A]. Then, we performed a luciferase assay using miR-30a-5p mimics that were cotransfected with luciferase reporters (containing wild-type or the mutant miR-30a-5p target sequence of mouse Sox4 mRNA 3′-UTR) into HEK-293 T cells. Compared with the negative control mimics, miR-30a-5p significantly inhibited the luciferase reporter activity in the cells with the wild-type mouse Sox4 mRNA 3′-UTR, but not the cells with the mutant mouse SOX4 mRNA 3′-UTR [Figure 6B]. In addition, our results indicated that upregulated hsa_circ_0020714 significantly promoted SOX4 expression in mouse lung cancer LLC cell line [Figure 6C]. To further determine the biological function of hsa_circ_0020714 on anti-PD-1 therapy resistance, we detected the anti-tumor effects of the PD-1 antibody in LLC-hsa_circ_0020714 xenograft *C57BL/6 *mice. Compared to that of the miR-30a-5p target sequence of mutant hsa_circ_0020714 group, the tumor growth increased in the LLC-hsa_circ_0020714 cells group which exhibited resistance to anti-PD-1 therapy and had a shorter survival time [Figure 6D-F].

## DISCUSSION

With the rapid development in the fields of bioinformatics and high-throughput sequencing techniques, the researchers have gradually deepened their understanding of circRNAs. Increasingly studies have verified that dysregulated circRNAs expression play critical roles in NSCLC immune evasion and resistance to anti-PD-1 immunotherapy. For example, our previous study demonstrated that forced circFGFR1 promotes resistance to anti-PD-1 treatment in NSCLC immunotherapy via sponging miR-381-3p to upregulate the expression of C-X-C motif chemokine receptor 4^[8]^. In another study, our results indicated that circMET overexpression enhanced NSCLC immune evasion via acting as a miR-145-5p sponge to upregulate CXCL3 expression^[10]^. Here, for the first time we reported that hsa_circ_0020714 plays a critical biological function in NSCLC immune evasion. We determined that hsa_circ_0020714 was upregulated in NSCLC tissues compared with paired adjacent nontumor tissues, and forced hsa_circ_0020714 expression was associated with a poor prognosis and resistance to anti-PD-1 immunotherapy in NSCLC patients. Mechanistically, hsa_circ_0020714 functions as an endogenous miR-30a-5p sponge to enhance SOX4 expression, subsequently inducing resistance to anti-PD-1 immunotherapy in NSCLC patients.

CircRNAs belong to a novel class of important ncRNAs involved in regulating various pathological and physiological processes. In recent years, they have attracted numerous research attention. Mounting evidence shows that dysregulated expression of circRNAs play a critical role in NSCLC progression and immune evasion^[8,10]^. In previous study, we proved that circMET overexpression can promote cancer cell proliferation, metastasis, and immune evasion in NSCLC^[10]^. In addition, we found that forced circFGFR1 expression promoted NSCLC progression and resistance to anti-PD-1 by sponging miR-381-3p^[8]^. In this study, we found that hsa_circ_0020714 was a critical oncogene that participates in immune evasion of NSCLC.

SOX4 is a member of the SOX family of transcription factors. Upregulated SOX4 expression participates in cancer progression, including proliferation, invasion, and migration^[11]^. In recent years, increasingly evidence has confirmed that the expression of SOX4 was regulated by a large class of ncRNA^[20-22]^. Our study identified SOX4 as a downstream important molecular of hsa_circ_0020714/miR-30a-5p axis in NSCLC cells. Recently, Bagati *et al.*^[13] ^found that forced SOX4 expression inhibits T cell-mediated anti-tumor immunity in triple-negative breast cancer. Furthermore, PD-1 blockade was verified to efficiently promote proliferation and expansion of CD8^+^ T cells in cancer model^[23]^. Over the past years, cancer immunotherapies have been demonstrated effectively therapeutic effect for some patients by blocking immune checkpoint receptors on T cells, restoring T cell-mediated cytotoxicity and recruiting more T cells into the tumor environment^[24]^. Here, our results also indicated that hsa_circ_0020714 could sponge miR-30a-5p to upregulate SOX4 expression in the NSCLC cells, thereby promoting NSCLC immune evasion and resistance to anti-PD-1 immunotherapy.

Taken together, our results demonstrate that hsa_circ_0020714 expression is upregulated in NSCLC tissues compared with the paired adjacent nontumor tissues. Mechanistically, hsa_circ_0020714 induces the immune evasion of NSCLC cells via sponging miR-30a-5p to upregulate SOX4 expression, which has been confirmed acted as a oncogene in several malignant tumors, including NSCLC. Therefore, blocking the hsa_circ_0020714/miR-30a-5p/SOX4-related pathway may effectively reverse resistance to anti-PD-1-based immunotherapy in NSCLC.

## DECLARATIONS

### Availability of data and materials

All data generated or analyzed in this study are included in this article.

### Copyright

© The Author(s) 2022.
